# First Draft Genome Sequence of a Member of the Genus *Mangrovibacter*, Isolated from an Aquaculture Farm in India

**DOI:** 10.1128/genomeA.01209-14

**Published:** 2014-11-20

**Authors:** Toms C. Joseph, Aswathy Mary Varghese, Anju Baby, Dinesh Reghunathan, V. Murugadas, K. V. Lalitha

**Affiliations:** Microbiology, Fermentation and Biotechnology Division, Central Institute of Fisheries Technology, Cochin, Kerala, India

## Abstract

*Mangrovibacter* sp. MFB070, a Gram-negative, facultatively anaerobic, nitrogen-fixing bacterium, was isolated from an aquaculture farm in Cochin, India. Here, we report the first draft genome sequence of a member of the genus *Mangrovibacter*, which may help us to elucidate the evolutionary status of this genus. The draft genome sequence of the *Mangrovibacter* sp. consists of 5,361,682 bp, encoding 4,971 predicted coding sequences in 57 contigs.

## GENOME ANNOUNCEMENT

*Mangrovibacter* sp. strain MFB070 was collected from a shrimp aquaculture farm in Cochin, India. It is a facultatively anaerobic, nitrogen-fixing bacterium representing a novel genus of the family *Enterobacteriaceae*, associated with the rhizosphere of mangrove-associated plants. Only one species, *Mangrovibacter plantisponsor*, with potential plant-beneficial properties was described (1). To date, there is no sequence information available on this genus. Therefore, we sequenced and analyzed the genome of *Mangrovibacter* sp. MFB070 to explore its genetic basis and its ability to fix nitrogen.

The draft genome of *Mangrovibacter* sp. strain MFB070 was performed on the Illumina MiSeq platform with a 2×250 paired-end run, after library preparation with the Nextera XT sample preparation kit (Illumina); 495,258 paired sequences were generated, for a total of >203.06 megabases and a mean length of 205 bases per read. Reads were analyzed and quality checked using FastQC (http://www.bioinformatics.babraham.ac.uk/projects/fastqc) and *de novo* assembled using Velvet (2), resulting in 57 contigs, the largest of which is 650,185 bp with an *N*_50_ of 222,077 bp. tRNA genes were predicted using the tRNAscan-SE program (3).

The genome sequence was annotated using NCBI GenBank and RAST (4) genome annotation servers. The draft genome sequence of *Mangrovibacter* sp. strain MFB070 is 5,361,682 bp, with a G+C content of ~ 50.4% and 4,971 predicted coding sequences, 20 rRNA operons, and 78 tRNA genes for all of the amino acids. The organism encodes for mdtABCD, the multidrug-resistant cluster that increases resistance to novobiocin and deoxycholate (5) and heavy-metal resistance including those for cobalt, cadmium, zinc, arsenic, and copper. Resistance of many bacteria to metal cations is predominantly based on metal-resistant determinants that contain genes for the RND (resistance, nodulation, and cell division protein family) protein (6). Several proteins were also identified that indicated the presence of phages in the bacteria. Genes involved in iron acquisition and metabolism, including siderophores, were also identified in the bacteria. The genome also contains ~35 open reading frames (ORFs) that match various phages. Twenty-one genes were involved in nitrogen fixation. The organism encodes for genes involved in nitrogen fixation, nitrosative stress, nitrate and nitrite ammonification, and ammonia assimilation.

The availability of the draft genome sequence of the *Mangrovibacter* sp. strain MFB070 will help us to elucidate the evolutionary status of this genus and also its role in nitrogen fixation.

### Nucleotide sequence accession number.

The draft genome sequence of *Mangrovibacter* sp. strain MFB070 is now available in GenBank database under accession no. JJMI00000000.
